# Randomized, Double-Blind, Placebo- and Positive-Controlled Crossover Study of the Effects of Omadacycline on QT/QTc Intervals in Healthy Subjects

**DOI:** 10.1128/AAC.00922-19

**Published:** 2019-09-23

**Authors:** Borje Darpo, Hongqi Xue, S. Ken Tanaka, Evan Tzanis

**Affiliations:** a ERT, Rochester, New York, USA; b Paratek Pharmaceuticals, Inc., King of Prussia, Pennsylvania, USA

**Keywords:** QTc study, acute bacterial skin and skin structure infections, community-acquired pneumonia, omadacycline

## Abstract

Omadacycline, an aminomethylcycline, is an antibiotic that is approved in the United States for once-daily intravenous (i.v.) and oral use for treatment of adults with acute bacterial skin and skin structure infections and community-acquired bacterial pneumonia. In this thorough QT study, the effects of a therapeutic (100 mg i.v.) dose and a supratherapeutic (300 mg i.v.) dose of omadacycline on the electrocardiogram were studied, with placebo and moxifloxacin as negative and positive controls. Omadacycline at these doses had no effect on the QTc interval. The largest mean placebo-corrected change-from-baseline QTcS (ΔQTcS) were 1.7 ms (90% confidence interval [CI], 0.06 to 3.30) and 2.6 ms (90% CI, 0.55 to 4.67), observed at 20 min and 2 h after the start of the infusion of 100 mg and 300 mg, respectively. Assay sensitivity was demonstrated with moxifloxacin, which caused clear prolongation of QTcS, with the largest mean placebo-corrected ΔQTcS of 9.8 ms at 1.5 and 2 h. With a linear exposure-response model, the estimated slope of the concentration-change-from-baseline QTcF (ΔQTcF) relationship was very shallow: 0.0007 ms per ng/ml (90% CI, 0.0000 to 0.0014). The possibility of an effect on placebo-corrected ΔQTcS exceeding 10 ms can be excluded at omadacycline concentrations in plasma of up to ∼8 μg/ml. Omadacycline had no effect on cardiac conduction (PR and QRS intervals) but caused an increase in heart rate of 16.8 beats per min at 35 min after the 100-mg dose and 21.6 beats per min at 50 min after the 300-mg dose.

## Abstract (precis)

Omadacycline, an aminomethylcycline, is an antibiotic that is approved in the United States for once-daily intravenous (i.v.) and oral use for treatment of adults with acute bacterial skin and skin structure infections and community-acquired bacterial pneumonia.

## INTRODUCTION

Omadacycline, the first aminomethylcycline antibiotic approved in the United States for once-daily intravenous (i.v.) and oral use in adults with acute bacterial skin and skin structure infections (ABSSSI) and community-acquired bacterial pneumonia (CABP), exhibits *in vitro* microbiological activity against Gram-positive and many Gram-negative aerobes and anaerobes and against atypical bacteria (1–3). Omadacycline is active against bacterial pathogens expressing the two forms of tetracycline resistance, i.e., efflux and ribosomal protection (4). In healthy volunteers who received either i.v. or oral doses, omadacycline exhibited a terminal elimination half-life of ∼17 to ∼18 h; peak drug concentrations in plasma were 1.8 μg/ml after a 100-mg i.v. dose, which is the dose being evaluated for treatment of community-acquired bacterial infections (5, 6). Omadacycline administered as a once-daily 100-mg i.v. or 300-mg oral dose demonstrated efficacy for the treatment of ABSSSI and CABP in phase 3 studies (7, 8) and is undergoing clinical development for urinary tract infections.

Cardiotoxic effects, primarily prolonged QTc interval, while rare, have been reported with fluoroquinolone, ketolide, and macrolide antibiotics (9–11). Because of the risk of drug-induced proarrhythmias, a thorough QTc study is typically required for drugs with systemic exposure, including antibiotics, as part of the clinical development program to fully characterize potential effects of the drug on electrocardiogram (ECG) parameters (12). In accordance with these requirements, this study evaluated the effect of therapeutic and supratherapeutic i.v. doses of omadacycline on QT/QTc intervals and the relationship between these intervals and plasma levels of omadacycline in healthy subjects.

## RESULTS

Eligible subjects were randomized to one of four treatment sequences. In the randomized sequences, each subject was to receive each of the following treatments: omadacycline 100 mg i.v. over 30 min, omadacycline 300 mg i.v. over 60 min, moxifloxacin 400 mg orally, or placebo. Placebo infusions to match omadacycline and placebo capsules to match moxifloxacin were used to maintain the study in a blind manner. Sixty-four eligible subjects were randomized, but two discontinued prior to receiving all four study treatments. An additional subject was not dosed with omadacycline 300 mg i.v. Thus, 61 subjects received all four study treatments. Data were analyzed per treatment for all the subjects receiving that treatment, regardless of whether they completed all four study treatments. The mean age of the randomized subjects was 27.6 years, 63% were male, 83% were Caucasian, mean body weight was 167 lb, and mean body mass index was 25.5 kg/m^2^.

### Pharmacokinetic analyses.

**FIG 1:**
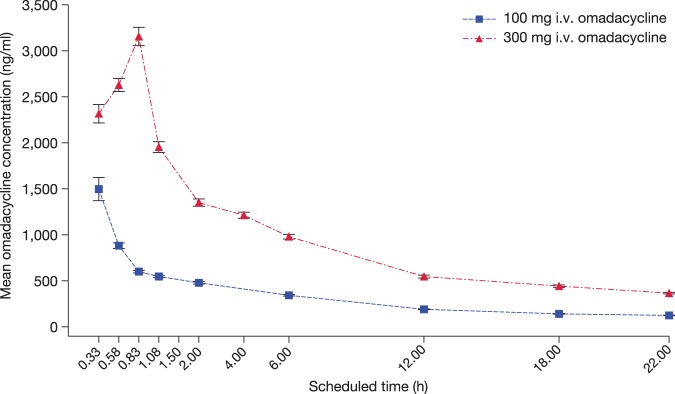
Plasma concentration-time curve for omadacycline 100- and 300-mg i.v. doses. Omadacycline infusions were given over 30 min for 100 mg and over 60 min for 300 mg. Peak drug concentrations in plasma were observed immediately after the end of the infusions. Data represent means ± standard errors.

Mean area under the concentration-time curve from 0 to 24 h (AUC_0–24_) and maximum concentration in plasma (*C*_max_) were dose proportional for omadacycline 100- and 300-mg i.v. doses (Table 1). *C*_max_ was observed at the end of the infusion for both doses (Fig. 1), reflecting the difference in infusion times (30 versus 60 min).

**TABLE 1:** Pharmacokinetic parameters after single 100-mg and 300-mg i.v. doses of omadacycline

Parameter^*a*^	Value(s) for indicated omadacycline dose	
	100 mg (*n* = 61)	300 mg (*n* = 61)
AUC_0–24_ (μg * h/ml)^*b*^	6.2 (1.1)	17.8 (2.9)
Range	4.4–9.2	9.5–25.3
CV (%)	17.3	16.3
*C*_max_ (μg/ml)^*b*^	1.4 (0.25)	3.3 (0.92)
Range	0.9–1.9	2.0–7.2
CV (%)	17.8	27.7
*T*_max_ (h)^*b*^	0.34 (0.04)	0.80 (0.11)
Range (h)	0.33–0.58	0.33–1.08

a AUC_0–24_, area under the concentration-time curve from 0 to 24 h; CV, coefficient of variation; *C*_max_, maximum concentration of drug in plasma; *T*_max_, time to maximum concentration of drug in plasma.

b Mean (standard deviation).

### ECG evaluations.

**FIG 2:**
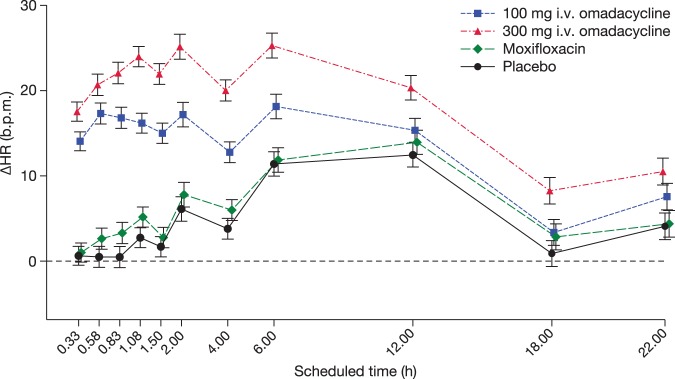
Change-from-baseline heart rate (ΔHR, bpm) across time points. Omadacycline caused a clear increase of ΔHR, which peaked near the end of the infusions. Bars show means ± 90% confidence intervals.

A similar diurnal pattern of change-from-baseline heart rate (ΔHR) was seen in the placebo and moxifloxacin periods, whereas omadacycline caused a clear effect on heart rate. The largest mean placebo-corrected ΔHR values seen with omadacycline were 16.8 beats per min (bpm) at 35 min postdose after the 100-mg dose and 21.6 bpm at 50 min after the 300-mg dose. The effect thereafter declined but remained above 5 bpm during 6 h after the 100-mg dose and for the full observation period up to 22 h in the highest dose group (Fig. 2).

Since the heart rate effect was pronounced, alternative ways of correcting the QT interval were explored. The mean correction factors for QTcP, QTcS, QTcI, and optimized QTcI (see Materials and Methods for definitions) were 0.34, 0.37, 0.37, and 0.33, which can be compared with Fridericia’s correction coefficient, 0.33. For QTcI, which included time points of graded exercise, the range of RR intervals (i.e., the time elapsed between two successive heartbeats) varied across subjects—between 160 and 720 ms—with a mean range of 375 ms. The observed range of RR intervals used to calculate the QTcI covered on-treatment RR ranges in 14 (22.2%) and 15 (24.6%) subjects in the omadacycline 100- and 300-mg i.v. groups, respectively. When day −1 baseline QT/RR data were used for derivation of optimized QTcI, the RR range by subject was substantially wider—between 800 and 2,995 ms—with a mean range of 1,175 ms. This RR range covered the on-treatment RR ranges in all subjects for both doses.

The abilities to remove heart rate dependence were compared among correction methods (Table 2). QTcS and QTcI resulted in the lowest mean sum of squared slopes (SSS), which was 0.0012 under drug-free conditions (placebo). Since QTcS resulted in lower mean SSS values on active doses, this correction method was selected as the primary endpoint.

**TABLE 2:** Comparison of the abilities of the correction methods to remove heart rate dependence^*a*^

Treatment	Slope estimate				
	QTcF	QTcI	Opt QTcI	QTcP	QTcS
100 mg i.v. omadacycline	0.0015	0.0018	0.0018	0.0014	0.0012
300 mg i.v. omadacycline	0.0017	0.0028	0.0021	0.0016	0.0020
Moxifloxacin	0.0019	0.0020	0.0019	0.0018	0.0015
Placebo	0.0017	0.0012	0.0016	0.0016	0.0012

a Slope estimate data represent means of squared individual slopes. i.v., intravenous; Opt, optimized.

**FIG 3:**
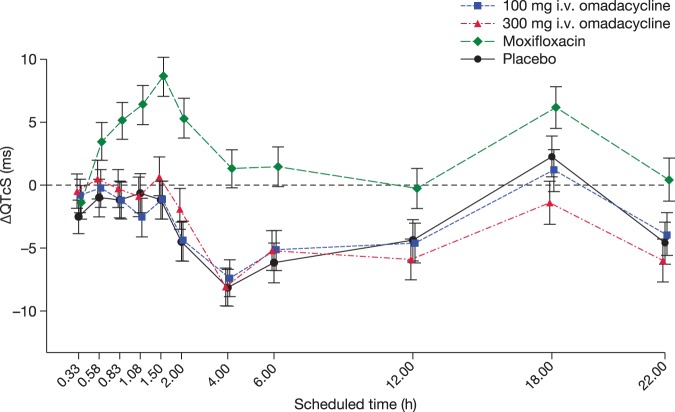
Change-from-baseline QTcS (ΔQTcS, ms) across time points. Mean ΔQTcS values determined after dosing with omadacycline closely followed the diurnal pattern seen in the placebo group, whereas 400-mg moxifloxacin caused clear QTc prolongation. Bars show means ± 90% confidence intervals.

Single doses of 100 and 300 mg omadacycline did not have an effect on the QTc interval. The pattern of mean change-from-baseline QTcS (ΔQTcS) closely followed that of placebo (Fig. 3). The largest mean placebo-corrected ΔQTcS values were only 1.7 ms (90% confidence interval [CI], 0.06 to 3.30) and 2.6 ms (90% CI, 0.55 to 4.67), observed at 20 min and 2 h after the start of the infusion of 100 and 300 mg, respectively (Table 3). After dosing with moxifloxacin, clear prolongation of QTcS was seen, with a largest mean placebo-corrected ΔQTcS of 9.8 ms at 1.5 and 2 h. All lower bounds of the 90% CI of placebo-corrected ΔQTcS values determined at 1.5, 2, and 4 h postdosing were above 5 ms, thereby demonstrating assay sensitivity. The results seen with other correction factors were similar to those determined for QTcS, with all mean placebo-corrected ΔQTc values below 2 ms and all upper bounds of the 90% CI below 5 ms. All mean placebo-corrected change-from-baseline QTcF (ΔQTcF) values were negative, and the upper bound of the 90% CI did not exceed 1.85 ms; for QTcI, the largest (positive) mean placebo-corrected ΔQTcI reached 1.6 ms (90% CI, −0.66 to 3.94) at 2 h, and for QTcP and optimized QTcI, the corresponding mean values were 0.0 ms (90% CI, −2.12 to 2.10) and −0.2 ms (90% CI, −2.37 to 1.97). There were no outliers in terms of QTcS above 480 ms or ΔQTcS above 60 ms.

**TABLE 3:** Placebo-corrected change-from-baseline QTcS^*a*^

Time point (postdose) and parameter	Value(s) (ms) for indicated treatment		
	Omadacycline dose		Moxifloxacin
	100 mg i.v.	300 mg i.v.	
20 min			
LS mean	1.7	2.0	1.1
SE	0.98	0.99	0.98
90% CI	(0.06 to 3.30)	(0.39 to 3.67)	(−0.49 to 2.76)
35 min			
LS mean	0.8	1.5	4.5
SE	1.15	1.17	1.15
90% CI	(−1.13 to 2.67)	(−0.44 to 3.41)	(2.56 to 6.37)
50 min			
LS mean	−0.1	0.9	6.3
SE	1.11	1.12	1.11
90% CI	(−1.91 to 1.74)	(−0.97 to 2.73)	(4.45 to 8.12)
65 min			
LS mean	−1.9	−0.3	7.0
SE	1.20	1.22	1.20
90% CI	(−3.86 to 0.10)	(−2.27 to 1.74)	(5.06 to 9.04)
1.5 h			
LS mean	0.0	1.9	9.8
SE	1.15	1.17	1.16
90% CI	(−1.90 to 1.92)	(−0.05 to 3.81)	(7.87 to 11.70)
2 h			
LS mean	0.1	2.6	9.8
SE	1.23	1.25	1.24
90% CI	(−1.98 to 2.09)	(0.55 to 4.67)	(7.73 to 11.82)
4 h			
LS mean	0.7	0.0	9.4
SE	1.13	1.15	1.14
90% CI	(−1.13 to 2.61)	(−1.87 to 1.93)	(7.56 to 11.33)
6 h			
LS mean	1.0	1.0	7.6
SE	1.23	1.24	1.23
90% CI	(−1.05 to 3.01)	(−1.07 to 3.03)	(5.59 to 9.65)
12 h			
LS mean	−0.2	−1.5	4.1
SE	1.25	1.26	1.25
90% CI	(−2.28 to 1.85)	(−3.59 to 0.59)	(2.05 to 6.19)
18 h			
LS mean	−1.1	−3.7	3.9
SE	1.30	1.31	1.30
90% CI	(−3.26 to 1.03)	(−5.86 to −1.51)	(1.72 to 6.02)
22 h			
LS mean	0.7	−1.4	5.0
SE	1.32	1.34	1.32
90% CI	(−1.49 to 2.88)	(−3.57 to 0.84)	(2.84 to 7.22)

a CI, confidence interval; i.v., intravenous; LS, least squares.

**FIG 4:**
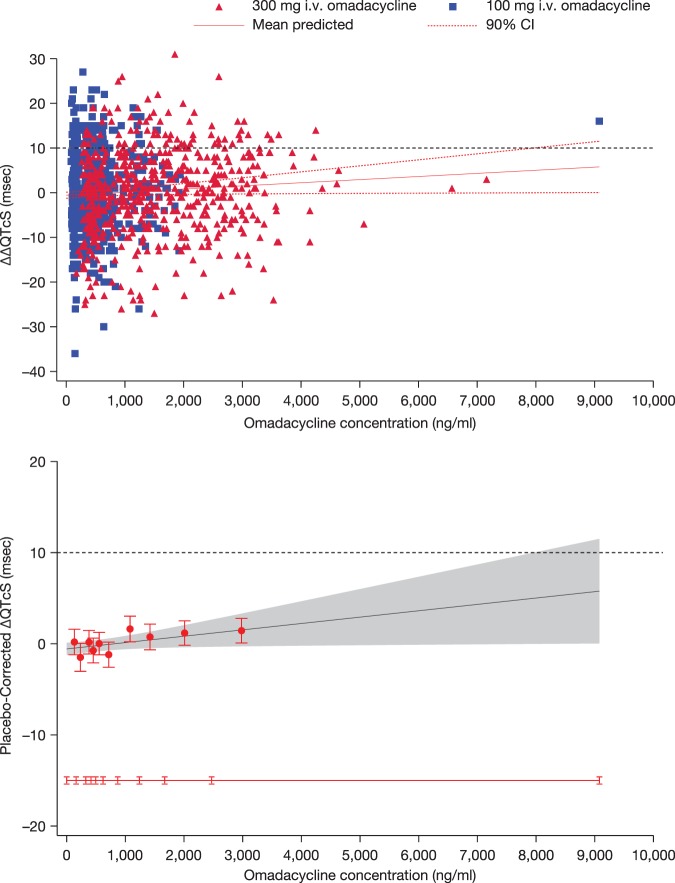
(Top panel) Scatter plot of placebo-corrected ΔQTcS versus omadacycline concentrations in plasma. The solid red line and dashed red lines denote the model-predicted mean placebo-corrected ΔQTcS with 90% CI. The blue squares and red triangles denote the pairs of observed omadacycline concentrations in plasma and predicted placebo-corrected ΔQTcS by subject for doses of 100 and 300 mg i.v., respectively. (Bottom panel) Model-predicted and observed placebo-corrected ΔQTcS across deciles of omadacycline concentrations in plasma. Red circles with vertical bars denote the observed mean placebo-corrected ΔQTcS with 90% CI displayed at the median drug concentration in plasma within each decile for omadacycline. The solid black line with gray shaded area denotes the model-predicted mean placebo-corrected ΔQTcS with 90% CI. The horizontal red line with notches shows the range of concentrations divided into deciles for omadacycline.

A linear exposure-response model provided an acceptable fit to the observed concentration and QT data. The relationship between the individual observed omadacycline concentrations and placebo-corrected ΔQTcS is visualized in the top panel in Fig. 4. The goodness-of-fit plot (Fig. 4, bottom panel) shows the mean placebo-corrected ΔQTcS (90% CI) within each omadacycline concentration decile and the model-predicted mean placebo-corrected ΔQTcS with 90% CI. From the plot, it can be seen that the predicted placebo-corrected ΔQTcS values are relatively close to the observed values, indicating that the proposed linear model provides an acceptable representation of the relationship between placebo-corrected ΔQTcS and omadacycline concentrations without accounting for hysteresis. The estimated slope of the concentration-ΔQTcS relationship was very shallow at 0.0007 ms per ng/ml (90% CI, 0.0000 to 0.0014), with a treatment effect-specific intercept of −0.6 ms (90% CI, −1.2 to 0.11). It can also be seen from the plot that the possibility of an effect on placebo-corrected ΔQTcS exceeding 10 ms can be excluded at omadacycline plasma levels up to ∼8 μg/ml.

### Safety/tolerability.

Adverse events were generally mild: more subjects had events of infusion-site pain following treatment with omadacycline 300 mg (11%) than with omadacycline 100 mg (6%), with rates for placebo and moxifloxacin of 0% and 2%, respectively. Infusion-site rash was reported in 5% of subjects following omadacycline 300 mg; no such events followed the other treatments. Headache was reported with an incidence of 3% to 7% following each treatment. There were no serious adverse events. One subject discontinued the study due to hives after receiving omadacycline 300 mg. This event was considered of moderate intensity and treated with oral diphenhydramine; it resolved ∼1 h after onset. No abnormal laboratory findings were reported.

## DISCUSSION

Prolongation of the QT interval is a well-documented effect of certain antibiotics, including fluoroquinolones, ketolides, and macrolides (10, 13, 14). For some of these drugs (e.g., telithromycin and azithromycin), postapproval warnings about potential cardiac toxicity were added to the product labeling (15). In addition, based on results of a thorough QTc study (16), telavancin carries a warning about QT interval prolongation in its product labeling. Thus, it is important to assess new antibiotics for their effects on ventricular repolarization.

Omadacycline is approved in the United States for treatment of adults with ABSSSI and CABP. For these indications, following an initial “loading” dose of 200 mg i.v. over 60 min or 100 mg i.v. over 30 min twice daily on day 1, omadacycline is administered once daily (either i.v. or orally), and the therapeutic i.v. dose is 100 mg (7, 8, 10). In healthy volunteers given a single 100-mg i.v. dose of omadacycline, mean *C*_max_ was 1.8 to 1.9 μg/ml (standard deviation, 0.4 to 0.7) (6, 17).

In this thorough QTc study, a therapeutic (100 mg i.v.) dose and a supratherapeutic (300 mg i.v.) dose of omadacycline had no effect on the QT interval. Since the possibility of an effect on placebo-corrected ΔQTcS above 10 ms could be excluded at all postdosing time points with both doses, the findings clearly represent negative QT study results as defined in the International Conference on Harmonisation E14 guidance (12). Using exposure-response analysis, as suggested in the recently revised E14 guidelines, the slope of the relationship between omadacycline concentrations in plasma and placebo-corrected ΔQTcS was very shallow—only 0.0007 ms per ng/ml. Consequently, estimating the effect throughout the full range of observed plasma levels, the possibility of an effect on placebo-corrected ΔQTcF exceeding 10 ms can be excluded up to omadacycline concentrations in plasma of ∼8 μg/ml, which is ∼4.4-fold above the mean *C*_max_ (∼1.8 μg/ml) in patients given the therapeutic dose of 100 mg i.v. over 30 min. In patients receiving the 200-mg i.v. dose, mean *C*_max_ levels would be somewhat higher (∼2.2 μg/ml), but the safety margin is still severalfold greater than that seen with concentrations that may lead to clinically relevant QT prolongation. It therefore seems highly unlikely that omadacycline will cause clinically concerning QT prolongation.

A clear and quite marked effect on heart rate was observed with mean peak effects of 16.8 and 21.6 bpm after the i.v. infusion of 100 mg and 300 mg, respectively. In such cases, it is important to evaluate which method for correction represents the best way to remove the heart rate dependence of the QTc interval. Tested in the manner proposed by a team from the U.S. Food and Drug Administration (18), the differences as shown by the SSS across the five methods applied to the data (QTcF, QTcS, QTcI, optimized QTcI, and QTcP) were small. As a consequence, the result of the QT evaluation was negative (i.e., upper bound of 90% CI of placebo-corrected ΔQTc of <10 ms) with all correction methods. Interestingly, QTcS and QTcI removed the heart rate dependence of the QT interval somewhat better than other methods using placebo data, but not consistently on omadacycline treatment. At the highest dose, 300 mg i.v., population-based methods (QTcF and QTcP), optimized QTcI and QTcS all worked better than QTcI. In our view, this observation argues for the importance of testing the ability of heart rate correction methods to remove the heart rate dependence of the QTc interval (19) and goes against the view that an individualized QTc method is by default always the best way to evaluate QT effect with drugs that have a pronounced heart rate effect (20).

When a heart rate effect of this magnitude is observed in healthy subjects, it becomes important to evaluate the effect in patients, to place the finding into a clinical context. It is not obvious that patients with infections who are febrile and experiencing stress would react the same way as healthy subjects when exposed to a drug that provides effective treatment for the infection. In a phase 3 study in adult patients with ABSSSI (7), no clinically significant heart rate changes were observed with omadacycline. In patients with CABP, the population was older and preexisting cardiovascular disease more frequent than were seen with the population in the infected-skin studies (21). Baseline heart rate was higher, and it seems reasonable to assume that the patients who were older with CABP—and who were, at times, hypoxic—were in greater distress than those with infected skin and skin structure. ECGs were recorded before and 30 to 90 min after the start of the 30-min infusion of the first and third doses. A small ΔHR (4.3 bpm) was observed after the first dose of omadacycline. With continued effective antibacterial therapy, no such effect was seen before or after the third dose, but the reduction of mean ΔHR was somewhat smaller than in the moxifloxacin group (1.8 and –1.1 versus –5.4 and –6.8 bpm, respectively). Importantly, throughout the omadacycline development program, very few outliers in terms of pronounced heart rate effects (patients with heart rate of >120 bpm and ΔHR of >15 bpm) have been observed, with no notable differences compared to active comparators. The observed increase in heart rate was small and transient and would not be expected to lead to adverse cardiac events. Such events, e.g., myocardial ischemia and episodes of decompensated heart failure, were few (0% to 0.3% and 0% to 0.8%, respectively) and occurred at rates similar to the observed incidence for comparators (0.3% to 0.5% for linezolid and moxifloxacin) (7, 8).

Nonclinical studies performed with omadacycline have demonstrated that the drug inhibits the M_2_ subtype of the muscarinic acetylcholine receptor, resulting in a nonadrenergic, vagolytic effect (22). In isolated rabbit sinus node preparations, omadacycline did not affect the intrinsic rate of the sinus node and, interestingly, reversed the bradycardia caused by cholinergic stimulation with carbamylcholine. These nonclinical results are consistent with the clinical observation that the heart rate effect of omadacycline is more pronounced in subjects with greater vagal tone and relatively low resting heart rate (i.e., in healthy subjects than in patients suffering from disease). A possible explanation for the difference observed to date between heart rate effects in healthy subjects and in patients with infections might be that in patients with active infections, the cholinergic impact on heart rate is attenuated through the effects of fever, dehydration, and systemic hypermetabolic manifestations of inflammation and distress with increased sympathetic tone.

In summary, these results demonstrate that omadacycline, at doses of 100 and 300 mg i.v., does not prolong the QTc interval. The observed heart rate effect in healthy subjects seems much less pronounced in patients with infections and is therefore not clinically concerning. The ability to place omadacycline in a category of low cardiac risk will be an important component to the overall benefit-risk assessment for this new antibiotic.

## MATERIALS AND METHODS

### Study design.

This was a single-dose, double-blind, randomized, four-way crossover study with an enrollment period extending from September 2008 to January 2009. The study was conducted in accordance with the ethical principles relating to biomedical research involving human subjects as adopted by the 18th World Medical Association General Assembly, Helsinki (1964) and subsequently amended. It was also conducted in accordance with local laws and regulations for the use of investigational therapeutic agents. The study protocol and all amendments were reviewed by the Institutional Review Board for the study center (PRACS Institute, Ltd.). The study investigator reviewed the study, and written information was provided to eligible subjects. Informed consent was obtained from each subject in writing before randomization.

Because the omadacycline i.v. solution had a yellow tint and the infusions were of different duration (30 or 60 min), all infusion bags were covered for anonymization and infusions were administered by personnel who did not participate in any other study procedures or assessments.

### Subject selection.

Healthy men and women aged 18 to 45 years were eligible, using standard criteria for clinical pharmacology studies. Women of childbearing potential had to use an effective birth control method from the time of screening until 30 days after the study; they also had to have had a negative pregnancy test at screening and on the day before dosing. Subjects were excluded for the following reasons: a history of allergy to any tetracycline or quinolone antibiotic; body weight of <45 or >100 kg; body mass index value of <18 or >30 kg/m^2^; systolic or diastolic blood pressure of >140 or >90 mm Hg, respectively; use of any investigational drug within 1 month prior to enrollment; clinically significant electrolyte abnormality; a history of cardiovascular disease, family history of QT prolongation, or any other medical condition or ECG abnormality that could interfere with the conducting of the study.

### Treatment groups.

Subjects were randomized to 1 of 4 treatment sequences, with the same treatments delivered in different orders. Subjects received an omadacycline therapeutic dose (100 mg i.v., infused over 30 min), an omadacycline supratherapeutic dose (300 mg i.v., infused over 60 min), a placebo infusion (negative control), and moxifloxacin (400 mg orally; active control). Moxifloxacin 400 mg or placebo capsules were given orally at the start of a 60-min placebo infusion. The omadacycline therapeutic dose corresponded to the proposed therapeutic dose for the phase 3 clinical trial of omadacycline in ABSSSI; the supratherapeutic dose was chosen because of good tolerability at this dose in phase 1 studies of omadacycline in healthy, young males.

### Study procedures.

The study was performed on an inpatient basis, and in each treatment period, subjects were admitted to the clinical site on day −2, 2 days before the day of dosing (day 1), and were discharged after completion of safety procedures on the day after dosing (day 2). The washout between consecutive study periods was ≥7 days. Study treatment was administered with subjects in the fasting state in the morning of day 1.

In treatment period 1, continuous 12-lead ECG data were recorded for 24 h on day −1, and subjects underwent a graded exercise test using a modified Bruce protocol with a target heart rate of >90 bpm 24 h and 25 min before dosing. On day 1 in all treatment periods, a continuous 12-lead ECG recording procedure was performed from 1.5 h before dosing to 24 h after dosing. The 12-lead ECGs were extracted in triplicate at three time points (1.5, 1.0, and 0.5 h) before dosing and at 20, 35, 50, and 65 min and 1.5, 2, 4, 6, 12, 18, and 22 h after dosing. ECG intervals were measured by a central ECG laboratory with a semiautomated technique.

Blood samples were obtained before dosing, at time points similar to those used for the postdose ECG extractions, and at 48, 72, and 96 h after the dose to measure omadacycline plasma levels and determine pharmacokinetic parameters, including *C*_max_, time to maximum concentration, and AUC_0–24h_. Blood samples were obtained 5 min after each time point indicated for ECG measurement.

### Statistical analysis.

Sample size was calculated based on the following assumptions: (i) the intrasubject standard deviation for change-from-baseline QTcF (ΔQTcF) would be 7 ms; (ii) the underlying QT effect for omadacycline would be 4 ms; (iii) ΔQTcF would be determined at 11 ECG time points per dose and at two doses. Based on these assumptions, a sample size of 50 subjects would provide more than 90% overall power to demonstrate that the upper bound of each one-sided 95% CI falls below 10 ms for up to 11 time points. Allowing for potential dropouts, a total of 64 subjects were to be randomized.

Baseline for each period was defined as the average of the measured QTc intervals from the three ECG time points (−1.5, −1.0, and −0.5 h) recorded before dosing in that period on day 1. The primary endpoint was the placebo-corrected change-from-baseline QTc (ΔQTc) determined using Fridericia’s correction (i.e., the placebo-corrected ΔQTcF) with QTcF = QT/RR^1/3^, unless a substantial peak heart rate (i.e., a mean placebo-corrected change-from-baseline HR [ΔHR] of >10 bpm in the “by time point” analysis) was observed in either of the two omadacycline treatment groups. In such cases, the following correction methods for QTc were to be explored and tested for their ability to remove the heart rate dependence.

### Method I.

Method I employed an individualized HR-corrected QT interval (QTcS) calculated from QT/RR data obtained at supine resting time points on day −1 in the first treatment period. Based on QT/RR pairs from all subjects, the QTcS correction coefficient was derived from a linear mixed-effects model as follows: log(QT) = log(*a*) + *b* × log(RR) (with gender included as a fixed effect and subject included as a random effect for both intercept and slope). The coefficient of log(RR) for each subject, *b_i_*, was then used to calculate QTcS for each subject as follows: QTcS = QT/RR^*b_i_*^.

### Method II.

Method II employed an individualized HR-corrected QT interval (QTcI) derived from QT/RR data obtained from a broader range of heart rates by also using ECG data extracted during time points of a graded exercise, in addition to the time points described above for day −1. Based on QT/RR pairs, the correction coefficient was derived, separately for each subject, from a linear regression model as follows: log(QT) = log(*a*) + *b* × log(RR). The coefficient of log(RR) for each subject, *b_i_*, was then used to calculate QTcI for that subject as follows: QTcI = QT/RR^*b_i_*^.

### Method III.

Method III employed a population HR-corrected QT interval (QTcP) derived from the same data as QTcS from all subjects. Based on QT/RR pairs from all subjects, QTcP was derived from a linear regression model as follows: log(QT) = log(*a*) + *b* × log(RR). The coefficient of log(RR), *b*, was then used to calculate QTcP for each subject as follows: QTcP = QT/RR*^b^*.

### Method IV.

Method IV employed an optimized HR-corrected QT interval (optimized QTcI) derived from a broader range of HRs by using all QT/RR data from day −1 of the first treatment period, i.e., QT/RR pairs from the full baseline 24-h recording. The QT/RR pairs from each subject were used for that subject’s individual correction coefficient, which was derived from a linear regression model as follows: log(QT) = log(*a*) + *b* × log(RR). The coefficient of log(RR) for each subject, *b_i_*, was then used to calculate QTcI2 for that subject as follows: QTcI2 = QT/RR^*b_i_*^.

For each method, the relationship between QTc and RR interval was then investigated using on-treatment data (omadacycline, moxifloxacin, and placebo) and a linear regression model as follows: QTc = *c* + *d* × RR. Mean QTc and RR values from all nominal time points (including predose) were used. The RR coefficient for each subject, *d_i_*, was then used to calculate the average SSS for each of the different QT-RR correction methods. The QTc method for which the average on-treatment slope value was closest to zero (i.e., the lowest average SSS value) for omadacycline and placebo was then selected as the primary endpoint (18).

The by-time-point analysis for QTc was based on a linear mixed-effects model with change-from-baseline QTc for the selected primary endpoint as the dependent variable; period, sequence, time (categorical), treatment (omadacycline, moxifloxacin, and placebo), and time-by-treatment interaction as fixed effects; and baseline QTc as a covariate. Subject was included as a random effect for the intercept. An unstructured covariance matrix was specified for the repeated measures at postdose time points for subject within the treatment period. From this analysis, the least-squares mean and two-sided 90% CIs were calculated for the contrast “omadacycline versus placebo” at each dose of omadacycline and each postdose time point, separately. For secondary endpoints (assay sensitivity and other ECG parameters), the same model was used. Assay sensitivity requirements were deemed to have been met if the placebo-corrected ΔQTc value was significantly above 5 ms at 1.5, 2, and 4 h postdose, controlling for multiplicity (23).

The relationship between the omadacycline concentrations in plasma and ΔQTc was investigated based on a linear mixed-effects model with ΔQTcF as the dependent variable, time-matched concentration of omadacycline as a continuous covariate (i.e., 0 for placebo), centered baseline QTc as an additional covariate, treatment (active = 1 or placebo = 0) and time as categorical factors, and a random intercept and slope per subject. The appropriateness of a linear model and, in cases of a QT effect in the by-time-point analysis, the presence of hysteresis (time delay between peak drug concentrations in plasma and peak QT effect) were tested.
